# 衍生多孔碳材料在固相微萃取中的应用研究进展

**DOI:** 10.3724/SP.J.1123.2022.06011

**Published:** 2022-10-08

**Authors:** Yixin KUANG, Suxin ZHOU, Yalan HU, Juan ZHENG, Gangfeng OUYANG

**Affiliations:** 1.中山大学化学学院, 广东 广州 510006; 1. School of Chemistry, Sun Yat-sen University, Guangzhou 510006, China; 2.湖南理工学院化学化工学院, 湖南 岳阳 414006; 2. School of Chemistry and Chemical Engineering, Hunan Institute of Science and Technology, Yueyang 414006, China

**Keywords:** 衍生多孔碳, 生物质炭, 金属有机框架, 固相微萃取, derived porous carbon, biomass carbon, metal-organic frameworks, solid-phase microextraction

## Abstract

固相微萃取是一种集采样、萃取、富集和进样于一体的样品前处理技术,其萃取效果与涂层材料密切相关。多孔碳材料具有比表面积大、多孔结构可控、活性位点多和化学稳定性好等优点,广泛应用于电池、超级电容器、催化、吸附和分离等领域,也是一种热门的用作固相微萃取探针的涂层材料。衍生多孔碳材料因种类丰富、可设计性强被广泛研究,研究主要集中在对衍生多孔碳材料的结构优化方面。但是衍生多孔碳材料在固相微萃取中的应用还存在如下问题:(1)共价有机框架衍生多孔碳材料的制备已取得较大进展,但将其应用于固相微萃取领域的研究仍较少;(2)有待进一步明确制备出的衍生多孔碳材料用作固相微萃取涂层表现出优异提取能力的机理;(3)有待进一步深入研究将衍生多孔碳材料用作固相微萃取涂层以实现对不同物理化学性质污染物的广谱高灵敏度分析。文章综述了近3年衍生多孔碳材料在固相微萃取中的应用研究,并展望了未来衍生多孔碳材料在固相微萃取中的研究前景。引用文献共56篇,主要来源于Elsevier。

## Abstract (en)

The concentrations of target analytes in samples are low, and complex matrices can lead to a variety of interferences. Therefore, it is important to pretreat the samples before analysis. Compared to the time-consuming, tedious, and environmentally unfriendly solvent-based sample pretreatment methods, pretreatment techniques based on adsorption have more promising applications. Adsorption-based pretreatment technologies include solid-phase extraction, dispersive solid-phase extraction, magnetic solid-phase extraction, and solid-phase microextraction. Among them, solid-phase microextraction integrates sampling, extraction, enrichment, and injection into a single step. It has the advantages of being solvent-free, highly efficient, time efficient, and labor efficient. The extraction efficiency of solid-phase microextraction is closely related to the coating materials. There are various types of coating materials, including metal-organic frameworks, covalent organic frameworks, molecular imprinted polymers, porous carbon materials and so on. Porous carbon materials include traditional porous carbon materials such as activated carbon, carbon nanotubes, carbon molecular sieves, and derived porous carbon materials. Given their advantages of large specific surface area, controllable porous structure, large number of active sites, as well as good physical and chemical stability, porous carbon materials have been widely used in batteries, supercapacitors, catalysis, adsorption, and separation. Porous carbon materials are also popular coating materials for solid-phase microextraction. In particular, derived porous carbon materials find widespread use given their variety and designability. Most of these materials are derived from biomass and metal-organic framework precursors. In addition, past studies have mainly focused on the structural optimization of derived porous carbon materials. However, the applications of derived porous carbon materials in solid-phase microextraction are restricted by the following problems. (1) The preparation of porous carbon materials derived from covalent organic frameworks has seen great progress. However, there are only a few studies on their applications in solid-phase microextraction. (2) The prepared-derived porous carbon materials have excellent extraction abilities as when applied to solid-phase microextraction coatings. However, there is less systematic and clear mechanism to explain it. (3) Most derived porous carbon materials when used as solid-phase microextraction coatings show nice extraction performance only for specific analytes such as polar or non-polar substances. Therefore, in this paper, the research progress of derived porous carbon materials in solid-phase microextraction over the past three years has been summarized, and future research prospects have been prospected. Covalent organic frameworks can be used as precursors to prepare derived porous carbon materials with a narrow pore size distribution and a large specific surface area. It is necessary to further develop porous carbon materials derived from covalent organic frameworks as solid-phase microextraction coatings. The specific mechanism underlying this extraction effect should also be clarified. In addition, it is necessary to develop high-performance derived porous coating materials for broad-spectrum and high-sensitivity analysis of pollutants with different physical and chemical properties. Therefore, hierarchical porous carbon materials should be widely studied in solid-phase microextraction because of their multimodal pore sizes. A total of 56 references are cited in this paper, most of which are from the Elsevier database.

固相微萃取(SPME)于1990年由Pawliszyn首次提出^[1]^,是一种集采样、萃取、富集和进样于一体^[1,2]^的样品前处理技术,绿色环保、高效且省时省力等优点使其广泛应用于环境^[3 ⇓ ⇓-6]^、生物^[6 ⇓-8]^、药物^[9,10]^和食品^[11,12]^分析中。SPME对目标分析物的萃取效果与分析物在样品基质和SPME涂层之间的分布平衡密切相关^[13]^,因此涂层材料在SPME中起着至关重要的作用。金属有机框架(metal-organic frameworks, MOFs)、共价有机框架(covalent organic frameworks, COFs)、分子印迹聚合物和多孔碳材料等已被用作SPME的涂层材料。

其中,多孔碳材料包括传统多孔碳材料以及生物质、MOFs、COFs、聚合物、金属氧化物和有机盐等衍生的多孔碳材料(见图1)。碳纳米管和石墨烯等传统多孔碳材料比表面积的局限和骨架结构的固定使得难以进一步提高其作为SPME涂层的提取性能^[14]^,而衍生多孔碳材料(derived porous carbon materials, DPCM)凭借比表面积大、多孔结构可控、活性位点多和化学稳定性好等优点成为极具前景的SPME涂层材料^[15,16]^。因此,本文综述了近3年来DPCM在SPME中的应用研究进展,并展望了未来DPCM在SPME中的研究前景。

**图1:**
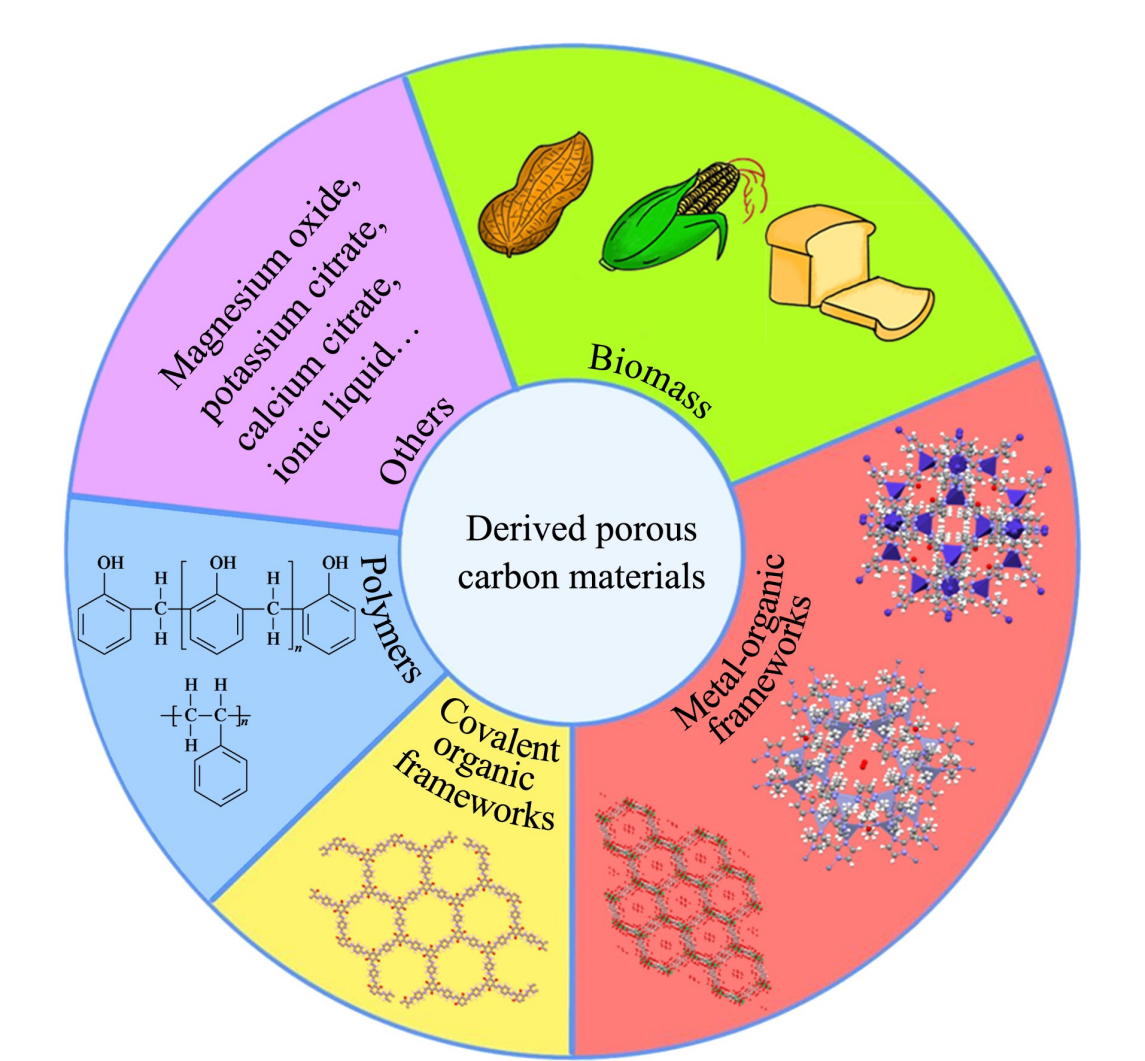
衍生多孔碳材料的来源

## 1 生物质衍生多孔碳材料

生物质来源丰富、可再生且绿色环保^[17]^,大多具有良好的吸附性能从而常被用作吸附剂和SPME涂层,但是仍存在富集效率较低的问题^[18]^。将生物质热解得到高度芳香化的生物质DPCM,应用于SPME领域可有效地解决该问题。Feng课题组在生物质DPCM用作SPEM涂层上研究较多,通过分别碳化蚕丝纤维^[19]^、棉纤维^[18]^和玉米芯^[20]^得到不同种生物质DPCM,然后将其填充到聚醚醚酮管中得到管内SPME器件,结合HPLC对真实水样中的多环芳烃(polycyclic aromatic hydrocarbon, PAHs)实现了痕量检测。此外,我们课题组采用10 ℃/min的速率升温至800 ℃并保持2 h的方法简单碳化花生壳,制得的SPME探针结合GC-MS实现了对环境水样中6种PAHs的检测^[21]^。

然而,生物质DPCM普遍存在比表面积小和内部孔道结构不发达的问题,因此为提高其吸附性能,通常采用活化的方式以获得更大的比表面积和丰富的孔结构。研究中多使用碳酸钾作为活化剂,例如Ji等^[22]^以含有大量蛋白质的石莼为原料,以碳酸钾为活化剂对其进行改性,制备了孔隙结构扩大、石墨化程度和疏水性改善的氮掺杂生物质DPCM,将其用作SPME探针可实现对水溶液中痕量氯苯(chlorobenzenes, CBs)的快速测定。类似地,我们课题组则采用碳酸钾在中等温度下对牛骨碳化后得到的富含杂原子的生物炭进行活化,以获得氧氮共掺杂的分级多孔生物炭,结果表明具有微孔、介孔和大孔分层孔隙结构的DPCM对极性和非极性分析物均具有优异的富集性能,因此将其用作SPME涂层可同时测定人尿中的邻二甲苯及其羟基代谢物^[23]^。

此外,将生物质DPCM与其他物质复合可赋予其在SPME领域更优异的性能。例如,与无机杂化材料的复合可以增加其表面积,Abolghasemi等^[24]^将层状双氢氧化物纳米片、定向勃姆石纳米线与山羊草茎衍生的生物质多孔碳复合,制备了具有三维分层开放结构的SPME探针,实现了对水样中三唑类、有机磷(organophosphorous, OPPs)和拟除虫菊酯类15种农药的痕量水平检测。而与MOFs复合能够综合MOFs对较小分子选择性化学吸附和生物质DPCM对较大分子较好的物理吸附的优势^[25]^。所以,Pasandideh等^[25]^使用面包生物质作为碳源,将面包衍生的多孔碳材料与Zn-MOF-5掺杂制备了Zn-MOF-5@面包碳氢多孔活性炭复合材料,成功用于不同基质中非甾类抗炎药的同时测定。

## 2 金属有机框架衍生多孔碳材料

MOFs凭借比表面积高、孔隙可调和可功能化修饰等优点被广泛研究。以MOFs为牺牲模板高温热解制备DPCM,在保持了MOFs原始形态的同时^[26,27]^,还具有优异的抗溶胀性、水稳定性和热稳定性^[28,29]^,是一类可应用于SPME领域的优异的涂层材料。

自2015年首次直接碳化铝基MOFs用作SPME涂层,并成功应用于提取水和土壤样品中的PAHs后^[30]^,多种MOFs衍生的多孔碳被用作SPME涂层材料。在其制备过程中,研究多采用单步碳化方式,将MOFs同时作为模板和碳前驱体^[31]^。Guo等^[13]^将ZIF-67在Ar/H_2_ (95%/5%, v/v)氛围下,350 ℃煅烧1.5 h,再以1 ℃/min的速率升温至700 ℃,保持4 h后逐渐降至室温。最后用0.5 mol/L的H_2_SO_4_处理以除去Co纳米颗粒,从而得到保留了ZIF-67结构且具有中空笼状结构的DPCM,并成功实现了对福建6个城市河水样品中多氯联苯(PCBs)的痕量检测。Yan等^[32]^采用在N_2_氛围下以10 ℃/min的速率从室温升至800 ℃并保持1 h直接碳化前驱体MOF-5-NH_2_,然后冷却至室温得到Zn和N共掺杂的DPCM,并成功用于牛肉干和鸭脖中极性酚的测定。

然而,直接碳化存在MOFs有序多孔结构的塌陷,进而导致金属位点团聚和孔隙率损失的问题^[33 ⇓-35]^。为解决这一问题,可以利用多孔模板的引入作为支撑^[29]^。例如,我们课题组将苯乙烯引入到MOF-74 (Ni)的通道中,通过其在碳化前的自聚合,碳化后可形成丰富的微孔,由此得到定制的具有多级孔隙和均匀分布Ni金属位点的MOF-74 (Ni)/PS-48 DPCM^[34]^,如图2所示。将其用作包覆SPME涂层可实现对人体体液中PAHs和OH-PAHs的高性能富集和高灵敏度分析。类似地,Xu等^[29]^选择具有大比表面积和多孔结构的纤维素纳米晶体作为MOF-74热解过程中的支撑材料,在有效保留了MOF-74的微孔结构的同时,还使得DPCM具有更大的孔体积,用作SPME涂层表现出对8种CBs分析物低至0.005~0.049 ng/L的检出限。此外,将石墨相氮化碳作为模板引入到NH_2_-MIL-125中,也可以防止热解过程中NH_2_-MIL-125孔隙的聚集和塌陷^[34]^。

**图 2:**
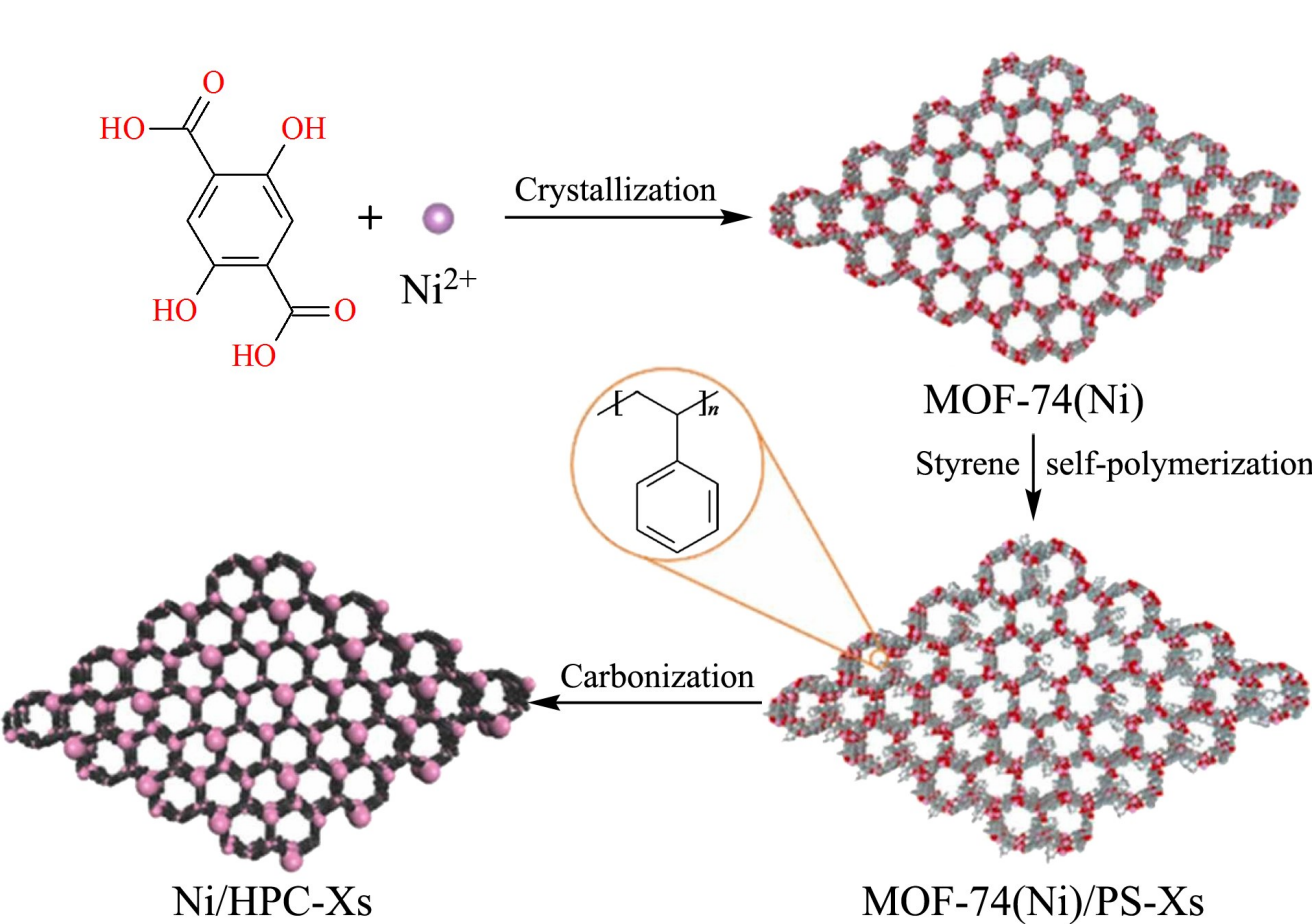
MOF-74 (Ni)/PS-48衍生多孔碳的合成^[34]^

在确保得到MOFs结构维持的DPCM的基础上,为了进一步实现MOFs制备的DPCM用作SPME涂层时具有更快的萃取效率、更高的灵敏度、更好的选择性或者是更广谱的分析范围,目前研究主要集中在对其微观结构的改变和功能化上。形貌的改变有利于获得更多的吸附位点,我们课题组首次将一种湿敏MOF MIL-101-NH_2_衍生的多孔碳材料用作SPME涂层^[36]^,并通过把氯化钴和硫脲封装到MIL-101-NH_2_中实现了从无定形碳形态到海胆状的形貌调节,从而提供了更大的比表面积、有效的吸附位点和尖锐边缘的传输通道,因此提高其提取能力和采样速度。除了特殊的海胆状形态,中空结构的基于MOFs的DPCM也可以加速传质过程,有利于解决固体和单调微孔结构对传质过程的限制带来的平衡时间较长和富集能力较低的问题^[37]^。另外还通过将制备的ZIF-8粉末经单宁酸刻蚀后形成中空结构^[38]^,然后在Ar气流下高温碳化得到ZIF-8衍生的纳米级中空碳纳米气泡。该中空碳纳米气泡具有超薄微孔壳和丰富的孔隙,因此在SPME的应用中,其空心内部结构对较小尺寸分析物表现出更高的吸附效率。进一步地,研究发现多壳空心结构比传统的中空纳米结构表现出更优越的效率^[39]^。因此,Hu等^[40]^采用单宁酸对ZIF-8进行刻蚀,然后热解得到双壳空心氧化锌/碳纳米立方体,用作SPME涂层可实现对复杂环境样品中非极性和极性污染物的低检出限分析。对基于MOFs的DPCM功能化则主要是通过杂原子掺杂,以此引入更多的吸附位点。例如,Li等^[41]^通过将直接碳化ZIF-67得到的DPCM经过35% HNO_3_氧化处理后获得了氧原子掺杂的多孔碳材料,将其用作一种新型SPME包覆材料。氧化处理产生大量的含氧官能团,进而实现了对极性分析物芳香胺(aromatic amines, AAs)的高效和高选择性富集。氧原子的掺杂可以提高对极性物质的相互作用力,类似地,氮原子的存在也会增加DPCM的极性,因此Pang等^[34]^合成了C-(C_3_N_4_@MOF)复合材料,作为SPME探针涂层从不同水果和蔬菜样品中萃取14种OPPs农药,且表现出对OPPs农药的高萃取效率。

此外,MOFs制备的DPCM作为SPME探针多采用溶胶凝胶法制备,原位生长MOFs操作简单且绿色环保^[42]^,因此原位制备基于MOFs的DPCM探针也成为SPME领域的研究热点。Wei等^[31]^通过把不锈钢丝浸入到2 mg/mL盐酸多巴胺中,在室温下搅拌12 h以获得聚多巴胺层以原位生长MOF-74,然后直接碳化首次实现了基于MOFs的DPCM的原位制备,并首次用于萃取有气味的有机污染物(odorous organic contaminants, OOCs)。此外,Guo等^[43]^则通过在0.05 mol/L苯胺和0.2 mol/L硝酸溶液中,在0.8 V恒电位下电解15 min以制备聚苯胺进而实现对不锈钢丝表面的改性,然后依靠共价相互作用原位生长MOF-67微晶,直接碳化得到氮掺杂石墨碳网络包覆的SPME探针,用于拟除虫菊酯的测定,并成功实现了葡萄和花椰菜样品中拟除虫菊酯的分析。

综上,MOFs衍生的多孔碳材料在SPME中的应用见表1。

**表1:** MOFs衍生多孔碳材料在固相微萃取中的应用

MOFs precursor	Properties	Matrices	Analytes	LODs/(ng/L)	Recovery/%	Ref.
ZIF-67	399 m^2^/g BET specific surface area	river water	PCBs	0.10-0.22	80.3-112.6	[13]
MOF-74	259 m^2^/g BET surface area, 0.35 cm^3^/g pore volume, thermal stability within 400 ℃	tap water, river water	CBs	0.005-0.049	93.2-116.8	[29]
MOF-74	298.6 m^2^/g surface area, 0.12 cm^3^/g pore volume, micropores ranging from 0.59 to 1.71 nm with an average size of 1.5 nm	tap water, freshwater, wastewater	OOCs	0.03-300	83.6-115.5	[31]
MOF-5-NH_2_	440.2 m^2^/g specific surface area, high hydrophilicity	beef jerky, duck neck	phenols	0.73-2.3	81.2-120.4	[32]
MOF-74	about 731 m^2^/g BET surface area, 1.07 nm pore size	human serum, artificial urine	PAHs, OH-PAHs	0.009-0.590	84.4-111.4	[33]
NH_2_-MIL-125	544 cm^3^/g surface area, 0.63 cm^3^/g total pore volume, 2.52 nm BJH median pore width, good thermal stability	apple, peach, pear, nectarine, plium, Chinese cabbage, baby cabbage, lettuce, pakchoi, oilseed rape	OPPs	0.23-7.5	82.6-118	[34]
MIL-101-NH_2_	1 μm diameter	pond water, river water	BTEX	0.08-0.36	85.3-113.3	[36]
ZIF-8	55 nm average diameter, 8.5 nm shell thickness, 556.0 m^2^/g BET surface area, 1.24 cm^3^/g pore volume	rainwater, pond water, river water	BTEX, PAHs	0.0017-0.0042 ng/L	95.3-117.1	[38]
ZIF-8	250 nm particle size, about 25 nm outer shell thickness	lake water, river water	BTEXCPs	0.14-0.561.10-2.84	92.6-97.092.5-99.5	[40]
ZIF-67	175.8 m^2^/g specific surface area, 1.10 nm micropore diameter, high hydrophilicity	polyethylene plastic food packaging bags	AAs	0.1-2.0	81.6-118.1	[41]
ZIF-67	333.75 m^2^/g BET specific area, 0.67 cm^3^/g pore volume	grape, cauliflower	pyrethroids	0.02-0.5	80.6-107.6	[43]
UiO-66-NH_2_	uniform mesopore, 583 m^2^/g specific surface area	pearl river water, pond water	phenols	0.21-1.7	84.5-108	[44]

ZIF: zeolitic imidazole framework; BET: Brunauer-Emmett-Teller; PCBs: polychlorinated biphenyls; CBs: chlorobenzenes; OOCs: odorous organic contaminants; PAHs: polycyclic aromatic hydrocarbon; MIL: Materials of Institute Lavoisier; BJH: Barret-Joyner-Halenda; OPPs: organophosphorous; BTEX: benzene toluene ethylbenzene & xylene; CPs: chlorophenols; AAs: aromatic amines; UiO: University of Oslo.

## 3 共价有机框架衍生多孔碳材料

与MOFs类似,COFs也凭借大比表面积、多孔结构、官能团丰富和可灵活设计等优点被开发作为SPME的高效涂层材料^[45]^。将COFs作为前驱体能够进一步得到具有高度贯穿孔结构、窄孔径分布和高比表面积的DPCM^[46]^,因此,Yan等^[47]^将TpPa-1该种COF用作自牺牲模板直接热解制备了DPCM,该材料具有5.62%的高含氮量、435.6 m^2^/g的高比表面积和3.9 nm的均匀孔径分布的优点,对PAHs表现出优异的提取能力。

## 4 聚合物衍生多孔碳材料

聚合物DPCM多以化学结构可调、拓扑形貌可控和易于加工的合成高分子为原料^[48]^。Zhang等^[49]^以石墨烯和酚醛树脂为前驱体进行碳化,制备得到导电性和热稳定性好、比表面积和孔容积高的三维石墨烯涂层用作电增强SPME工作电极,可提高对双酚A的提取效率,并成功用于对3种热敏纸中双酚A的分析。在该研究中,酚醛树脂的添加是作为交联剂诱导石墨烯结构的优化,以解决石墨烯纳米片倾向于团聚的问题。我们课题组^[44]^则以酚醛树脂为前驱体制备有序介孔碳,同时通过与UiO-66-NH_2_的复合以实现Zr和N的共掺杂,制备得到的SPME探针表现出对苯酚更快的吸附速率和更高的提取能力。

此外,如图3所示,Chen等^[5]^选择形貌和孔隙结构可控的聚苯乙烯,通过直接碳化超交联后的F/Cl官能化聚苯乙烯纳米球得到F/Cl官能化的微孔碳,碳化赋予其更多的微孔和更高的表面积,将其用作SPME涂层可充分利用丰富的微孔作为吸附位点,实现F官能化微孔碳对二甲苯高达59374的富集因子,F/Cl官能化微孔碳对萘的富集效率提高。

**图3:**
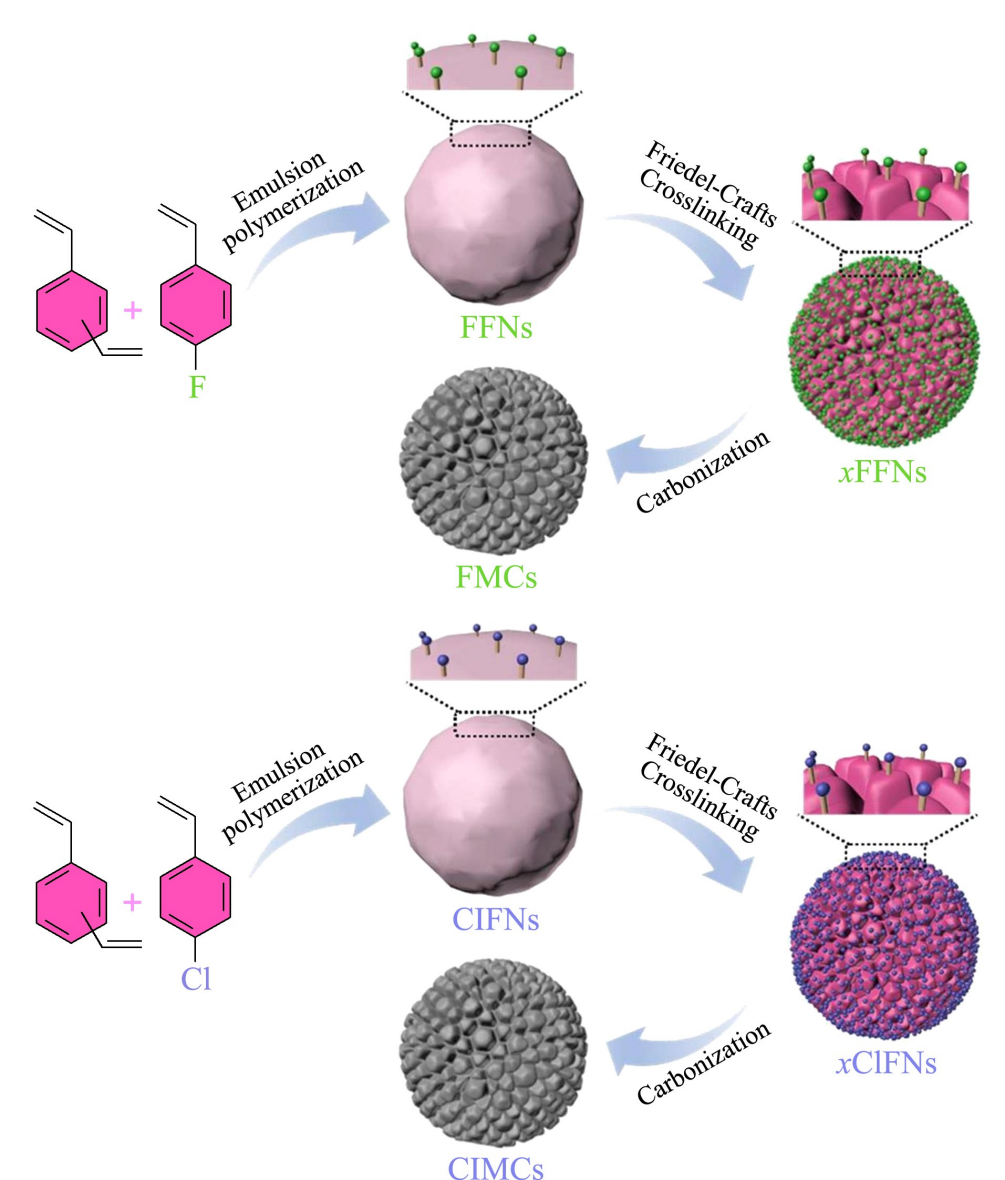
具有可变微结构的功能纳米球的制备^[5]^

## 5 其他多孔碳材料

除了常见的生物质、MOFs、COFs和聚合物等DPCM外,金属氧化物和有机盐衍生的多孔碳材料应用于SPME涂层也有一定的研究。

### 5.1 金属氧化物衍生多孔碳材料

金属氧化物纳米结构特殊的形貌和孔隙结构可提供大比表面积^[50]^,也是应用于SPME领域极具潜力的涂层材料。然而,近几年对金属氧化物得到的DPCM用作SPME涂层的研究较少。仅有我们课题组采用自组装的方式制备了具有三维分层花状结构的乙醇酸镁球体,然后直接碳化得到形态保持的多级孔隙MgO&C复合材料^[50]^,将其用作SPME涂层对实际河水样品中PAHs进行检测时,所建立的方法对PAHs表现出0.01~0.20 ng/L的低检出限。

### 5.2 有机盐衍生多孔碳材料

利用碳化碱性金属有机盐制备DPCM的过程中,由于金属蒸汽可起到造孔作用,因此碱性金属有机盐既是碳前驱体也是活化剂^[51]^,通过其自活化可避免化学活化法带来的低产量、活化剂有毒、有腐蚀性以及活化剂去除的问题,相关研究还处于起步阶段。Cheng等^[52]^首次将柠檬酸钾和柠檬酸钙的混合物在100 mL/min的超纯N_2_气氛下以5 ℃/min的速率升温至850 ℃并保持1 h对其进行碳化和活化,合成了具有高达3270 m^2^/g的表面积和1.79 cm^3^/g孔体积的新型多峰多孔碳。同时可以通过改变柠檬酸钾和柠檬酸钙混合物的比例实现对孔径分布的可控调节,用作一种新型SPME探针对CBs和PCBs具有很好的提取能力。而在其另一个研究工作中^[53]^,基于有机碱盐柠檬酸钙自活化的基础上,进一步将柠檬酸钙自活化后的产物与氢氧化钾混合对其进行二次活化。这种双级活化方式可以产生具有丰富介孔和微孔的多孔碳,同时比表面积高达2638.09 m^2^/g。用作SPME涂层材料表现出比商用针高48.5倍的萃取能力,并应用于实际水样中痕量CBs的测定。

此外,离子液体作为一种由阴阳离子组成的特殊的有机盐,具有绿色环保、高热稳定性和结构可调性等优点^[54,55]^,是用作SPME的优秀涂层材料。目前,离子液体在SPME领域的研究多为开发新的聚离子液体以提高其稳定性和吸附性能,较少将离子液体碳化得到DPCM用作SPME涂层。Dong等^[56]^采用将模板剂离子液体添加到间苯二酚-甲醛的缩聚过程中,经高温碳化得到了具有介孔交联结构的离子液体碳气凝胶复合材料,同时采用浓硫酸/硝酸氧化法进行处理得到羧基化的离子液体碳气凝胶。羧基功能化为其提供了丰富的活性位点,将其制备成SPME探针表现出对6种四环素(tetracyclines, TCs)优异的提取效果,并成功应用于鸡蛋和家禽养殖场废水样品中TCs残留物的检测。

## 6 总结和展望

总的来说,应用于SPME涂层的DPCM主要以生物质和MOFs为原料,采用直接碳化的方式制备,而更多的则是在对“明星材料”MOFs衍生的多孔碳材料的研究上,未来在COFs衍生的多孔碳材料方面还有较大的研究空间。同时,由于SPME的提取性能很大程度上取决于涂层材料的比表面积和孔隙结构,因此目前DPCM在SPME领域的研究多集中在对材料结构的优化方面,以获得更大的比表面积和更丰富的多级孔结构。

然而,研究中多将制备出的DPCM用作SPME涂层的出色提取能力归因于其与分析物之间的多种相互作用,如尺寸选择性、微孔填充、*π-π*堆积和疏水性等^[53]^,却没有提出明确的机理。同时,研究制备的多孔碳材料包覆SPME涂层大多表现出对非极性或极性分析物优异的提取性能,现有研究致力于实现对不同物理化学性质污染物的广谱高灵敏度分析,但仍有待更深入的研究。
